# Supplementary material for: Transient oxytocin signaling primes the development and function of excitatory hippocampal neurons
Source: eLife. 2017 Feb 23;6:e22466. doi: 10.7554/eLife.22466 (PMC5323041; doi:10.7554/eLife.22466)
Supplement: Supplementary file 1. — The Table provides an overview of all the electrophysiological experiments performed in this paper. The conditions used for each experiment are reported together with the related figures. Data are expressed as mean ± SEM. Fs: figure supplement. DOI: http://dx.doi.org/10.7554/eLife.22466.023 [file elife-22466-supp1.docx]

**SUPPLEMENTARY FILE 1**

**Table 1. Summary of the synaptic transmission analysis.**

| **AUTAPTIC CULTURES** | | | | | | | | | | | | | | | | | | | | |
| --- | --- | --- | --- | --- | --- | --- | --- | --- | --- | --- | --- | --- | --- | --- | --- | --- | --- | --- | --- | --- |
| **PSC responses** | | **Glutamatergic neurons**  **(Hippocampus)** | | | | | | | | | **GABAergic neurons**  **(Hippocampus)** | | | | | | | | | **Related figure** |
|  | | **Ctrl** | | **Oxt 1d** | | | **Oxt 3d** | | | | **Ctrl** | | | | **Oxt 1d** | | | **Oxt 3d** | |  |
| PSC (nA) | | 3.15±0.23  (n=116) | | 1.97±0.16  (n=110) | | | 2.21±0.18  (n=107) | | | | 3.9±0.56  (n=43) | | | | 4.1±0.53  (n=51) | | | 4.1±0.47  (n=47) | | Fig. 2c |
| Sucrose response (nC) | | 0.40±0.04  (n=85) | | 0.23±0.02  (n=72) | | | 0.27±0.02  (n=75) | | | | 1.5±0.19  (n=38) | | | | 1.49±0.25  (n=38) | | | 1.34±0.14  (n=39) | | Fig. 2d |
| Pvr (%) | | 10.4±0.75  (n=85) | | 11.0±0.96  (n=72) | | | 10.4±1.12  (n=75) | | | | 22.1±1.79  (n=38) | | | | 24.4±1.84  (n=38) | | | 22.8±2.04  (n=39) | | Fig. 2e |
| mPSC amplitude (pA) | | 18.8±0.96  (n=57) | | 18.9±0.96  (n=50) | | | 20.3±1.11  (n=51) | | | | 23.1±2.33  (n=20) | | | | 26.0±3.11  (n=23) | | | 25.3±1.94  (n=26) | | Fig. 2j |
| mPSC frequency (Hz) | | 7.1±0.85  (n=57) | | 4.4±0.55  (n=50) | | | 4.2±0.68  (n=51) | | | | 2.6±0.90  (n=20) | | | | 2.3±0.56  (n=23) | | | 2.3±0.66  (n=26) | | Fig. 2k |
| 100 μM glutamate response (nA) | | 0.98±0.08  (n=76) | | 0.65±0.051  (n=89) | | | 0.61±0.04  (n=96) | | | | - | | | | - | | | - | | Fig. 2g |
| 3 μM GABA response (nA) | | - | | - | | | - | | | | 0.94±0.19  (n=26) | | | | 1.06±0.16  (n=25) | | | 0.97±0.13  (n=30) | | Fig. 2h |
|  | | **Ctrl**  **(E18)** | | **Oxt 1d (E18)** | | | **Oxt 3d**  **(E18)** | | | |  | | | |  | | |  | |  |
| PSC (nA) | | 2.69±0.20  (n=66) | | 1.96±0.18  (n=60) | | | 1.79±0.22  (n=60) | | | | - | | | | - | | | - | | Fig. 2-fs. 3g |
| Sucrose response (nC) | | 0.33±0.040  (n=53) | | 0.19±0.022  (n=50) | | | 0.19±0.032  (n=51) | | | | - | | | | - | | | - | | Fig. 2-fs. 3i |
| Pvr (%) | | 10.86±0.87  (n=53) | | 9.46±0.98  (n=50) | | | 11.62±1.21  (n=51) | | | | - | | | | - | | | - | | Fig. 2-fs. 3h |
| 100 μM glutamate response (nA) | | 1.57±0.13  (n=58) | | 1.04±0.09  (n=49) | | | 1.08±0.09  (n=46) | | | | - | | | | - | | | - | | Fig. 2-fs. 3j |
|  | | **Ctrl** | | **U73122** | | | **U73122+Oxt** | | | |  | | | |  | | |  | |  |
| PSC (nA) | | 1.8±0.21  (n=49) | | 2.2±0.26  (n=38) | | | 2.1±0.26  (n=39) | | | | - | | | | - | | | - | | Fig. 2-fs. 4c |
| Sucrose response (nC) | | 0.19±0.03  (n=31) | | 0.22±0.03  (n=30) | | | 0.18±0.03  (n=30) | | | | - | | | | - | | | - | | Fig. 2-fs. 4d |
| Pvr (%) | | 12.8±2.6  (n=31) | | 13.61±1.5  (n=30) | | | 14.23±1.7  (n=30) | | | | - | | | | - | | | - | | Fig. 2-fs. 4e |
| mPSC amplitude (pA) | | 19.9±0.94  (n=36) | | 22.2±1.04  (n=33) | | | 22.1±1.31  (n=30) | | | | - | | | | - | | | - | | Fig. 2-fs. 4j |
| mPSC frequency (Hz) | | 3.6±0.46  (n=36) | | 3.5±0.79  (n=33) | | | 3.77±0.68  (n=30) | | | | - | | | | - | | | - | | Fig. 2-fs. 4k |
| 100 μM glutamate response (nA) | | 0.74±0.06  (n=38) | | 0.77±0.09  (n=33) | | | 0.71±0.12  (n=30) | | | |  | | | |  | | |  | | Fig. 2-fs. 4i |
|  | | **Ctrl** | | **Oxt 7d** | | | **Oxt 7-9d** | | | |  | | | |  | | |  | |  |
| PSC (nA) | | 2.9±0.41  (n=33) | | 3.1±0.39  (n=26) | | | 2.6±0.36  (n=31) | | | | - | | | | - | | | - | | Fig. 2-fs. 2c |
| Sucrose response (nC) | | 0.35±0.07  (n=27) | | 0.39±0.08  (n=24) | | | 0.37±0.06  (n=22) | | | | - | | | | - | | | - | | Fig. 2-fs. 2d |
| Pvr (%) | | 9.9±1.6  (n=27) | | 9.5±1.4  (n=24) | | | 9.3±2.0  (n=22) | | | | - | | | | - | | | - | | Fig. 2-fs.2e |
| mPSC amplitude (pA) | | 19.2±1.03  (n=26) | | 21.3±1.12  (n=24) | | | 21.6±1.05  (n=25) | | | | - | | | | - | | | - | | Fig. 2-fs. 2j |
| mPSC frequency (Hz) | | 2.6±0.38  (n=26) | | 2.8±0.42  (n=24) | | | 2.4±0.42  (n=25) | | | | - | | | | - | | | - | | Fig. 2-fs. 2k |
| 100 μM glutamate response (nA) | | 0.92±0.20  (n=22) | | 0.91±0.16  (n=27) | | | 0.80±0.10  (n=28) | | | | - | | | | - | | | - | | Fig. 2-fs.2h |
|  | | **Ctrl** | | **PTX** | | | **PTX+Oxt** | | | |  | | | |  | | |  | |  |
| Relative PSC | | 1±0.09  (n=36) | | 1.16±0.18  (n=27) | | | 0.69±0.10  (n=28) | | | | - | | | | - | | | - | | Fig. 2-fs. 5b |
| Relative sucrose response | | 1±0.10  (n=31) | | 1.06±0.18  (n=24) | | | 0.61±0.11  (n=21) | | | | - | | | | - | | | - | | Fig. 2-fs.5d |
| Relative 100 μM glutamate | | 1±0.06  (n=48) | | 1±0.13  (n=33) | | | 0.8±0.09  (n=25) | | | | - | | | | - | | | - | | Fig. 2-fs. 5f |
| **PSC responses** | | **Glutamatergic neurons**  **(Hippocampus)** | | | | | | | | | | | | | | | | | | |
|  | | ***Oxtr^Vn/Vn^*** | | | | | | ***Oxtr^Vn/Vn^* +Oxt 1d** | | | | | | | | ***Oxtr^Vn/Vn^* +Oxt 3d** | | | | **Related Figure** |
| PSC (nA) | | 1.88±0.27  (n=18) | | | | | | 1.65±0.22  (n=21) | | | | | | | | 1.54±0.18  (n=18) | | | | Fig. 1-fs. 3g |
| Sucrose response (nC) | | 0.25±0.03  (n=16) | | | | | | 0.21±0.02  (n=19) | | | | | | | | 0.18±0.02  (n=16) | | | | Fig. 1-fs. 3h |
| Pvr (%) | | 6.37±0.83  (n=16) | | | | | | 6.23±1.10  (n=19) | | | | | | | | 7.11±0.80  (n=16) | | | | Fig. 1-fs. 3i |
| 100 μM glutamate response (nA) | | 1.49±0.15  (n=18) | | | | | | 1.33±0.17  (n=18) | | | | | | | | 1.16±0.14  (n=16) | | | | Fig. 1-fs. 3j |
|  | | **Ctrl** | | | **ATO 1d** | | | | **ATO+Oxt 1d** | | | | **ATO3d** | | | | **ATO+Oxt 3** | | |  |
| PSC (nA) | | 1.9±0.20  (n=48) | | | 1.9±0.29  (n=25) | | | | 1.8±0.20  (n=41) | | | | 1.8±0.19  (n=41) | | | | 1.9±0.22  (n=33) | | | Fig. 2-fs. 1c |
| Sucrose response (nC) | | 0.20±0.03  (n=31) | | | 0.21±0.02  (n=24) | | | | 0.21±0.05  (n=24) | | | | 0.18±0.03  (n=22) | | | | 0.17±0.02  (n=24) | | | Fig. 2-fs. 1d |
| Pvr (%) | | 9.5±0.99  (n=31) | | | 9.5±1.3  (n=24) | | | | 8.4±1.6  (n=24) | | | | 8.4±1.5  (n=22) | | | | 8.7±0.94  (n=24) | | | Fig. 2-fs. 1e |
| mPSC amplitude (pA) | | 19.4±1.11  (n=30) | | | 17.2±2.77  (n=22) | | | | 19.2±1.16  (n=24) | | | | 17.4±0.73  (n=24) | | | | 18.4±1.06  (n=23) | | | Fig. 2-fs. 1j |
| mPSC frequency (Hz) | | 3.1±0.74  (n=30) | | | 3.4±0.84  (n=22) | | | | 3.4±0.51  (n=24) | | | | 3.3±0.63  (n=24) | | | | 3.2±0.83  (n=23) | | | Fig. 2-fs. 1k |
| 100 μM glutamate response (nA) | | 0.56±0.06  (n=30) | | | 0.55±0.11  (n=22) | | | | 0.58±0.05  (n=26) | | | | 0.54±0.09  (n=27) | | | | 0.49±0.05  (n=22) | | | Fig. 2-fs. 1i |
| **PSC responses** | | **GABAergic neurons**  **(Striatum)** | | | | | | | | | | | | | | | | | | |
|  | | **Ctrl** | **Oxt 1d** | | | **Oxt 3d** | | | | **EGFP+**  **Oxt 1d** | | | | **Oxtr+**  **Oxt 1d** | | | **EGFP+**  **Oxt 3d** | | **Oxtr+**  **Oxt 3d** | **Related Figure** |
| PSC (nA) | | 4.1±0.4  (n=41) | 4.2±0.44  (n=43) | | | 3.8±0.42  (n=47) | | | | 4.6±0.51  (n=32) | | | | 2.8±0.45  (n=29) | | | 5.2±0.61  (n=44) | | 2.6±0.38  (n=34) | Fig. 3d |
| Sucrose response (nC) | | 1.6±0.2  (n=28) | 1.6±0.15  (n=33) | | | 1.4±0.14  (n=35) | | | | 2.0±0.25  (n=26) | | | | 1.3±0.15  (n=23) | | | 1.7±0.23  (n=37) | | 1.0±0.14  (n=28) | Fig. 3e |
| Pvr (%) | | 30.4±3.0  (n=28) | 27.8±2.7  (n=33) | | | 27.3±2.7  (n=35) | | | | 24.5±2.3  (n=26) | | | | 21±2.4  (n=23) | | | 30±2.3  (n=37) | | 25.2±2.1  (n=28) | Fig. 3f |
| 3 μM GABA response (nA) | | 1±0.12  (n=24) | 0.8±0.12  (n=28) | | | 0.8±0.07  (n=29) | | | | 1.7±0.23  (n=24) | | | | 1.1±0.13  (n=20) | | | 1,7±0,18  (n=35) | | 1.1±0.1  (n=30) | Fig. 3g |
| n= number of analyzed cells. Data from *Oxtr^Vn/Vn^* are derived from three independent experiments from a single preparation while all other data are derived from at least 3 independent preparations. | | | | | | | | | | | | | | | | | | | | |
| **MASS CULTURES AND SLICES** | | | | | | | | | | | | | | | | | | | | |
|  | ***Oxtr^+/+^* neurons** | | | | | | | | | | | ***Oxtr^-/-^* neurons** | | | | | | | | **Related Figure** |
| sEPSC interval (ms) | 266±49 (n=11) | | | | | | | | | | | 149±31 (n=7) | | | | | | | | Fig. 5j |
| sEPSCs amplitude (pA) | 19.7±2.1 (n=11) | | | | | | | | | | | 30.9±1.9 (n=7) | | | | | | | | Fig. 5k |
| n= number of analyzed cells. Data are derived from 3 independent preparations. | | | | | | | | | | | | | | | | | | | | |
|  | **Ctrl neurons** | | | | | | | | | | | **TGOT-treated neurons** | | | | | | | | **Related Figure** |
| sEPSC interval (ms) | 108±9 (n=16) | | | | | | | | | | | 471±60 (n=11) | | | | | | | | Fig. 5-fs. 2g |
| sEPSC amplitude (pA) | 40.5±2.2 (n=16) | | | | | | | | | | | 29.5±4.6 (n=11) | | | | | | | | Fig. 5-fs. 2f |
| n= number of analyzed cells. Data are derived from 3 independent preparations. | | | | | | | | | | | | | | | | | | | | |
|  | ***Oxtr^+/+^* slices** | | | | | | | | | | | ***Oxtr^Vn/Vn^* slices** | | | | | | | | **Related Figure** |
| mEPSC amplitude (pA; CA1) | 7.86±0.2 (n=24) | | | | | | | | | | | 8.40±0.23 (n=18) | | | | | | | | Fig. 7b |
| mEPSC amplitude (pA; CA3) | 10.5±0.70 (n=23) | | | | | | | | | | | 13.13±1.20 (n=19) | | | | | | | | Fig. 7b |
| mEPSC frequency (Hz; CA1) | 0.28±0.04 (n=24) | | | | | | | | | | | 0.53±0.12 (n=18) | | | | | | | | Fig. 7c |
| mEPSC frequency (Hz; CA3) | 0.82±0.16 (n=23) | | | | | | | | | | | 1.4±0.23 (n=19) | | | | | | | | Fig. 7c |
| mIPSC amplitude (pA; CA1) | 26.8±1.78 (n=25) | | | | | | | | | | | 25.96±2.31 (n=18) | | | | | | | | Fig. e |
| mIPSC amplitude (pA; CA3) | 23.5±1.60 (n=25) | | | | | | | | | | | 26.33±2.62 (n=19) | | | | | | | | Fig. 7e |
| mIPSC frequency (Hz; CA1) | 4.42±0.39 (n=25) | | | | | | | | | | | 5.41±0.36 (n=18) | | | | | | | | Fig. 7f |
| mIPSC frequency (Hz; CA3) | 5.84±0.31 (n=25) | | | | | | | | | | | 5.96±0.41 (n=19) | | | | | | | | Fig. 7f |
| n= number of analyzed cells. Data are derived from juvenile mice (P15-26). For mIPSCs and mEPSCs 4 and 5 animals were analyzed per genotype. | | | | | | | | | | | | | | | | | | | | |
| γ-oscillations maximum power (μV^2^/Hz) | 263.3±35.74 (n=8) | | | | | | | | | | | 131.3± 31.54 (n=6) | | | | | | | | Fig. 7h |
| γ-oscillations power (μV^2^/Hz) | 132.2±17.5 (n=8) | | | | | | | | | | | 56.48±16.22 (n=6) | | | | | | | | Fig. 7i |
| γ-oscillations Frequency (Hz) | 35.22±1.28 (n=8) | | | | | | | | | | | 33.76± 1.53 (n=6) | | | | | | | | Fig. 7j |
| n= number of juvenile mice (P15-26) analyzed. For each mouse, 6-8 different slices were recorded. | | | | | | | | | | | | | | | | | | | | |
