# Supplementary material for: Transient oxytocin signaling primes the development and function of excitatory hippocampal neurons
Source: eLife. 2017 Feb 23;6:e22466. doi: 10.7554/eLife.22466 (PMC5323041; doi:10.7554/eLife.22466)
Supplement: Supplementary file 2. — The Table provides an overview of all the morphological experiments performed in this paper. The conditions used for each experiment are reported together with the related figures. Data are expressed as mean ± SEM. Fs: figure supplement. DOI: http://dx.doi.org/10.7554/eLife.22466.024 [file elife-22466-supp2.docx]

**SUPPLEMENTARY FILE 2**

**Table 2. Summary of the morphological analysis.**

| **AUTAPTIC CULTURES** | | | | | | | | | | | | | | |
| --- | --- | --- | --- | --- | --- | --- | --- | --- | --- | --- | --- | --- | --- | --- |
|  | **Hippocampal cultures** | | | | | | | | | | | | | **Related Figure** |
|  | **Ctrl** | | | | **Oxt 1d** | | | | | **Oxt 3d** | | | |  |
| Number of total intersection (25-80 μm) | 110.8±9.5 (n=40) | | | | 82.03±6.2 (n=40) | | | | | 75.73±6.6 (n=40) | | | | Fig. 1c |
| Number of VGLUT punta/cell | 228±22.4 (n=47) | | | | 149.6±18.9 (n=33) | | | | | 156.8±19.8 (n=45) | | | | Fig. 1e |
| Number of PSD-95 puncta/cell | 175±26.0 (n=47) | | | | 102.8±12.3 (n=33) | | | | | 111.1±12.26 (n=45) | | | | Fig. 1f |
| Mander’s coefficient | 0.89±0.004 (n=15) | | | | 0.87±0.01 (n=15) | | | | | 0.97±0.01 (n=15) | | | | Fig. 1g |
|  | **Ctrl (E18)** | | | | **Oxt 1d (E18)** | | | | | **Oxt 3d (E18)** | | | |  |
| Number of total intersection (25-80 μm) | 102.4±5.30 (n=70) | | | | 78.10±4.78 (n=69) | | | | | 70.87±4.48 (n=69) | | | | Fig. 2-fs 3c |
|  | ***Oxtr^Vn/Vn^*** | | | | ***Oxtr^Vn/Vn^* +Oxt 1d** | | | | | ***Oxtr^Vn/Vn^* +Oxt 3d** | | | |  |
| Number of total intersection (25-80 μm) | 70.90±3.94 (n=41) | | | | 79.75±6.10 (n=40) | | | | | 3.95±4.76 (n=39) | | | | Fig. 1-fs 3c |
|  | **Ctrl** | | | | **U73211** | | | | | **U73211+Oxt** | | | |  |
| Number of total intersection (25-80 μm) | 109±7.88 (n=34) | | | | 97.03±11.07 (n=33) | | | | | 93.31±7.58 (n=32) | | | | Fig. 2-fs. 4n |
|  | **Ctrl** | | **ATO 1d** | | | **ATO+Oxt 1d** | | **ATO3d** | | | | **ATO+Oxt 3** | |  |
| Number of total intersection (25-80 μm) | 89.12±4.9  (n=50) | | 74.89±8.0  (n=44) | | | 86.54±7.7  (n=50) | | 76.93±6.26  (n=49) | | | | 92.27±6.90  (n=49) | | Fig. 1j |
|  | **Striatal neurons** | | | | | | | | | | | | | **Related Figure** |
|  | **Ctrl** | **Oxt 1d** | | **Oxt 3d** | | | **EGFP+**  **Oxt 1d** | | **Oxtr+**  **Oxt 1d** | | | **EGFP+**  **Oxt 3d** | **Oxtr+**  **Oxt 3d** |  |
| Number of total intersection (25-80 μm) | 74.2±6.9  (n=36) | 81.64±8.8  (n=30) | | 78.06±6.37  (n=31) | | | 87.11±4.8  (n=43) | | 64.96±7.5  (n=21) | | | 91.36±5.3  (n=44) | 64.21±6.0  (n=20) | Fig. 3k |
| Number of VGAT punta/cell | 146.2±19.23  (n=27) | 151.6±17.6  (n=29) | | 162.2±18  (n=27) | | | - | | - | | | - | - | Fig. 3-fs. 1b |
| Number of gephyrin puncta/cell | 69.89±6.8  (n=27) | 64.83±6.9  (n=29) | | 75.13±6.8  (n=27) | | | - | | - | | | - | - | Fig. 3-fs. 1c |
| n= number of analyzed cells. Data from *Oxtr^Vn/Vn^* are derived from two independent experiments from a single preparation while all other data are derived from at least 3 independent preparations. | | | | | | | | | | | | | | |
| **MASS CULTURES AND SLICES** | | | | | | | | | | | | | | |
|  | ***Oxtr^+/+^* neurons** | | | | | | | ***Oxtr^-/-^* neurons** | | | | | | **Related Figure** |
| VGAT puncta density/10 μm | 1.04±0.09 (n=177) | | | | | | | 0.81±0.07 (n=177) | | | | | | Fig.5b |
| VGLUT1 puncta density/10 μm | 1.12±0.07 (n=200) | | | | | | | 1.65±0.09 (n=200) | | | | | | Fig.5c |
| NL2 cluster density/10 μm | 2.35±.019 (n=76) | | | | | | | 1.74±0.16 (n=76) | | | | | | Fig.5f |
| Gephyrin cluster density/10 μm | 2.58±0.14 (n=135) | | | | | | | 1.85±0.10 (n=132) | | | | | | Fig.5f |
| GABAγ2 cluster density/10 μm | 2.05±0.12 (n=135) | | | | | | | 2.18±0.13 (n=130) | | | | | | Fig.5f |
| NL1 cluster density/10 μm | 1.76±0.10 (n=90) | | | | | | | 2.22±0.13 (n=90) | | | | | | Fig.5g |
| PSD95 cluster density/10 μm | 2.07±0.13 (n=133) | | | | | | | 2.90±0.15 (n=129) | | | | | | Fig.5g |
| GluA1 cluster density/10 μm | 2.82±0.16 (n=132) | | | | | | | 3.34±0.18 (n=132) | | | | | | Fig.5g |
| n= number of analyzed dendrites. Data are derived from 3 independent preparations. | | | | | | | | | | | | | | |
| GAD65/67-positive neurons (%) | 11.56±1.48 (n=3) | | | | | | | 12.14±0.35 (n=3) | | | | | | Fig. 5-fs. 1b |
| Parvalbumin-positive neurons (%) | 8.05±2.53 (n=3) | | | | | | | 9.79±1.87 (n=3) | | | | | | Fig. 5-fs. 1d |
| Data are derived from 3 independent neuronal preparations. | | | | | | | | | | | | | | |
|  | **Ctrl neurons** | | | | | | | **TGOT-treated neurons** | | | | | | **Related Figure** |
| VGAT puncta density/10 μm | 0.94±0.06 (n=198) | | | | | | | 1.31±0.01 (n=192) | | | | | | Fig. 5-fs. 2c |
| VGLUT1 puncta density/10 μm | 1.54±0.17 (n=213) | | | | | | | 0.97±0.06 (n=200) | | | | | | Fig. 5-fs. 2c |
| NL2 cluster density/10 μm | 2.14±0.14 (n=206) | | | | | | | 2.62±0.17 (n=212) | | | | | | Fig. 5-fs. 2d |
| NL1 cluster density/10 μm | 1.70±0.07 (n=192) | | | | | | | 1.45±0.07 (n=175) | | | | | | Fig. 5-fs. 2d |
| n= number of analyzed dendrites. Data are derived from 3 independent preparations. | | | | | | | | | | | | | | |
|  | **Ctrl neurons** | | | | | **Oxt 1d-treated neurons** | | | | | **Oxt 3d-treated neurons** | | | **Related**  **Figure** |
| % of pyknotic nuclei (E18) | 29.15±2.11 | | | | | 37.85±2.76 | | | | | 32.03±32.03 | | | Fig. 1-fs 1b |
| % of pyknotic nuclei (P0) | 27.96±2.27 | | | | | 32.70±4.82 | | | | | 29.33±2.45 | | | Fig. 1-fs 1b |
| Data are derived from 3 independent experiments from 3 different preparations. Each treatment assessed between 4000-5000 cells. | | | | | | | | | | | | | | |
|  | ***Oxtr^+/+^* slices** | | | | | | | ***Oxtr^-/-^* slices** | | | | | | **Related Figure** |
| Parvalbumin-positive neurons/0.18 mm^2^ | 17.44±2.64 (n=3) | | | | | | | 16.02±1.50 (n=3) | | | | | | Fig. 5-fs. 1f |
| n=number of 3-month old male mice examined. For each mouse 3 sections were analyzed. | | | | | | | | | | | | | | |
|  | ***Oxtr^+/+^* slices** | | | | | | | ***Oxtr^Vn/Vn^* slices** | | | | | | **Related Figure** |
| Number of apical branches (CA1) | 5.52±0.64 (n=34) | | | | | | | 7.55±0.73 (n=38) | | | | | | Fig. 6b |
| Number of basal neurites (CA1) | 3.69±0.29 (n=34) | | | | | | | 5.48±0.23 (n=38) | | | | | | Fig. 6c |
| Length of apical shaft (CA1) | 55.10±6.23 (n=34) | | | | | | | 58.56±7.73 (n=38) | | | | | | Fig. 6d |
| Density of filopodia (per 50 μm, CA1) | 3.78±0.46 (n=34) | | | | | | | 7.88±0.85 (n=38) | | | | | | Fig. 6e |
| Number of primary neurites (CA3) | 4.35±0.19 (n=17) | | | | | | | 6.52±0.36 (n=17) | | | | | | Fig. 6g |
| n=number of cells analyzed. Around 10 littermates (P7) from *Oxtr^Vn/+^* interbreedings were analyzed out of 7 operated *Oxtr^Vn/+^* females. | | | | | | | | | | | | | | |
| Number of apical branching point (0-300 μm, CA1) | 14.00±1.35 (n=9) | | | | | | | 19.31±1.62 (n=16) | | | | | | Fig. 8d |
| Number of basal branching point (0-150 μm, CA1) | 14.30±1.77 (n=10) | | | | | | | 18.60±2.16 (n=15) | | | | | | Fig. 8f |
| Number of apical branching point (0-100 μm, CA3) | 6.85±0.70 (n=14) | | | | | | | 10.75±1.18 (n=12) | | | | | | Fig. 8h |
| Number of basal branching point (0-150 μm, CA3) | 16.23±1.47 (n=13) | | | | | | | 22.73±2.85 (n=11) | | | | | | Fig. 8j |
| n=number of cells analyzed. Data were obtained from 6 different animals per genotype. | | | | | | | | | | | | | | |
| Total spine number (apical) | 1922±269 (n=5) | | | | | | | 2566±338 (n=4) | | | | | | Fig. 8l |
| Total spine number (basal) | 921.5±239 (n=4) | | | | | | | 1954±173 (n=3) | | | | | | Fig. 8l |
| Spine density/10 μm (apical) | 9.26±0.46 (n=5) | | | | | | | 9.88±0.65 (n=4) | | | | | | Fig. 8m |
| Spine density/10 μm (basal) | 8.30±0.75 (n=4) | | | | | | | 9.79±0.87 (n=3) | | | | | | Fig. 8m |
| Spine number (stubby, apical) | 600.4±106.35 (n=5) | | | | | | | 775.5±152.9 (n=4) | | | | | | Fig. 8n |
| Spine number (thin, apical) | 649.6±55.4 (n=5) | | | | | | | 976.5±256.8 (n=4) | | | | | | Fig. 8n |
| Spine number (mushroom, apical) | 672.0±169 (n=5) | | | | | | | 814±192.2 (n=4) | | | | | | Fig. 8n |
| Spine number (stubby, basal) | 298±68.5 (n=4) | | | | | | | 383.6±68.7 (n=3) | | | | | | Fig. 8o |
| Spine number (thin, basal) | 420.7±116.1 (n=4) | | | | | | | 1025±127.9 (n=3) | | | | | | Fig. 8o |
| Spine number (mushroom, basal) | 211±59.6 (n=4) | | | | | | | 545±59.31 (n=3) | | | | | | Fig. 8o |
| n=number of cells analyzed. Data were obtained from 3 different animals per genotype | | | | | | | | | | | | | | |
